# Factors associated with epileptic seizure of cavernous malformations in the central nervous system in West China

**DOI:** 10.12669/pjms.295.3681

**Published:** 2013

**Authors:** Cheng Huang, Ming-Wan Chen, Yang Si, Jin-Mei Li, Dong Zhou

**Affiliations:** 1 Cheng Huang, Department of Neurology, West China Hospital of Sichuan University, China.; 2 Ming-Wan Chen, West China School of Medicine, Sichuan University, China.; 3 Yang Si, Department of Neurology, West China Hospital of Sichuan University, China.; 4 Jin-Mei Li, Department of Neurology, West China Hospital of Sichuan University, China.; 5 Dong Zhou, Department of Neurology, West China Hospital of Sichuan University, China.

**Keywords:** Cerebral cavernous malformation, Epilepsy, Neurosurgery, Risk factors

## Abstract

***Objective: ***To explore the factors associated with preoperative and postoperative epileptic seizure in patients with cavernous malformations (CMs).

***Methods: ***A total of 52 consecutive patients from January 2009 to June 2011 who underwent surgical treatment in West China Hospital of Sichuan University due to CMs and confirmed by histopathology were retrospectively reviewed.Patients were divided into two groups (epilepsy-group and non-epilepsy group) according to clinical presentation. Other clinical data, treatment procedure, and follow-up information were collected. Engel classification was used to evaluate seizure outcome.

***Results: ***Low birth weight, temporal lobe involvement and cortical lesion showed significant difference between two groups (*p*=0.017, 0.003 and 0.025 respectively). Cortical lesion highly increased risk for preoperative epileptic seizure (OR=10.48; 95% CI 1.61-68.23). After a mean follow-up of 2.1 years, 77.8% of epileptic patients achieved Engel class I. Temporal lobe involvement, lesion size < 2.5cm and surgery within one year of symptom onset were found associated with better seizure outcome (*p*=0.016, 0.012 and 0.050). Temporal lobe involvement significantly decreased the risk for postoperative epileptic seizure (OR=0.038; 95% CI 0.002-0.833). Application of ECoG made no significant difference to seizure outcome (*p*=0.430). Most patients need continuing medication therapy after surgery.

***Conclusion: ***Surgical treatment of patient with CMs is satisfactory in most cases and temporal lobe involvement usually predict favourable postoperative seizure outcome whether under the monitoring of ECoG or not. Thus, epileptic patients with CMs should be considered for surgical treatment especially when cortical brain layer or temporal lobe was involved.

## INTRODUCTION

Cavernous malformations (CMs) are common vascular malformations in the central nervous system which usually consist of enlarged thin-wall blood vessels without neural tissue. The prevalence of CMs in population is around 0.4-0.8% and seizures is common clinical presentation (23-50% of cases).^1^ Other main symptoms include headache (6-52%), neurological deficits (20-40%) and hemorrhage (9-56%).^1^ There are a number of studies discussing clinical manifestation, diagnostic procedure, treatment and surgical outcome of CMs.^2^^-^^4^

However, these studies occasionally distinguished arteriovenous malformations (AVMs) and CMs and usually discussed about the global risk brought by CMs.^3^^,^^5^ Few studies considered about the specific risk factors for developing epileptic seizure in patients with CMs especially preoperative. Age, cortical site, family history, pre-, peri- and postnatal factors were reported as independent predictors of symptomatic seizures.^5^ Other factors such as alcohol and tobacco addiction may increase the risk of epileptic seizure as well according to clinical practice.^5^ Factors influencing postoperative seizure occurrence are still in dispute. Thus, in order to explore the factors associated with preoperative and postoperative epileptic seizure in patients with CMs, we retrospectively analyzed the clinical data of 52 consecutive patients diagnosed with CMs and surgically treated in West China Hospital of Sichuan University during the period from January 2009 to June 2011.

## METHODS

A total of 52 consecutive patients diagnosed with CMs who underwent surgical treatment in Department of Neurosurgery, West China Hospital of Sichuan University from January 2009 to June 2011 were retrospectively studied. Inclusion criteria were patients with CMs confirmed by pathology after surgery. Patients who were clinically diagnosed with CMs on the evidence of neuroimaging but confirmed as other type of vascular malformations such as AVMs were excluded.

We assigned patients with preoperative epilepsy into epilepsy group (E-Group) and those with headache, focal neurological deficits (FND) or intracranial hemorrhage (ICH) into non-epilepsy group (NE-Group). We defined epilepsy as presentation of seizure not symptomatic of ICH caused by CMs at least two times, and ICH as symptomatic events associated with evidence of intracranial blood on neuroimaging and FND as symptoms of neurologic dysfunction related to the anatomic site of CMs but without presence of epileptic seizure. We grouped CMs locations into 4 types: supratentorial lobar (frontal, parietal, temporal, occipital), supratentorial deep area (limbic, thalamus, hypothalamus, callosal, basal ganglia, and choroidal), infratentorial(brain-stem and cerebellum), spine, or multiple (if the patient had at least two CMs). The size of CMs was measured as the maximum diameter of the CM.

The following information was collected during the review of medical records: gender, age, disease course, neurological manifestation at admission (seizure, headache, FND or ICH), diagnostic procedures, neuroimage character, anatomic location, size of CMs, surgical procedures, histopathology results. In order to explore the risk factors of seizure in patients with CMs, we also spared no efforts to collect following data: family history of epileptic seizure, pre- and peri- and postnatal factors, history of tobacco and alcohol use.

Follow-up information was obtained by out-patient and telephone interviews. The surgical outcome of patients with epilepsy was classified according to the Engel’s classification into class I, completely seizure free, seizure free for at least two years, auras only, or convulsions with drug withdrawal only; class II, rare seizure (≤2 seizures per year); class III, worthwhile improvement; class IV, no significant improvement or worse. For patients without seizure, the surgical outcome was classified into following four classes according to patients’ subjective feelings: class I, significant improvement; class II, improvement; class III, no significant improvement; class IV, worsen.

The analysis of risk factors for epilepsy in patients with CMs was estimated by calculating odds ratios (OR), with 95% confidence intervals (95% CI). The chi-square test and two-tailed t-test were performed when appropriate. The significance level was set at 0.05. Variables, which would potentially predict increasing risk for epilepsy, were then evaluated by logistic regression model.

## RESULTS

The study consisted of 52 patients: 31 (59.6%, 31/52) in E-Group and 21 (40.4%, 21/52) in NE-Group (Table-I). Of the 31 patients with epilepsy, the predominant seizure type was secondarily generalized seizure (74.2%, 22/31), followed by complex partial seizure (29.0%, 9/31), simple partial seizure (3.2%, 1/31). Twenty patients (39.2%) were determined as refractory epilepsy.

The sex ratio, mean age at admission and mean age at symptom onset were found similar in two groups. Symptoms of CMs included epilepsy (59.6%, 31/52), headache (17.3%, 9/52), focal neurological deficits (19.2%, 10/52), intracranial hemorrhage (3.5%, 2/52). There are 25 in E-Group and 18 in NE-Group clearly clarified the data of size of tumor. We considered only the supratentorial lesions when calculating the OR value for cortical lesion. The reference category is patients without involvement of that specific lobe (either unilobar or multilobar) when calculating the OR value for specific lobe.

**Table-I:** General information of patients with CMs (52 cases)**.**

	*E-Group*	*NE-Group*	*p value*
Number	31	21	-
Gender (M:F)	2.44	1.10	0.242
Male	22	11	-
Female	9	10	-
Age (y, ± SD)	36.2 ± 13.4	36.3 ± 12.1	0.963
Mean age at onset, y, ± SD	31.3 ± 13.4	34.0 ± 13.8	0.473

***Factors associated with preoperative epileptic seizure: ***In our study, the factors seen significantly associated with preoperative epilepsy were low birth weight (*p*=0.017), temporal lobe involvement (*p*=0.003) and cortical lesion (*p*=0.025) (Table-II). No significant association was found between other pre-, peri- and post-natal factors (complication of pregnancy, low gestational age, complication of delivery, febrile seizure, psychomotor retardation, history of encephalitis) and preoperative epilepsy separately (shown in Table-II). However, there were more patients with at least one of these risk factors in E-Group than NE-Group (14 vs. 3) (*p*=0.020). Mean age at onset, family history of epilepsy, history of injury, operation, tobacco and alcohol using were not seen associated with an increased risk for epilepsy (shown in Table-II). In the multivariate analysis, the cortical lesion still showed a high risk for epilepsy (OR=10.48; 95% CI 1.61-68.23) while the pre- and peri- and postnatal factors (OR=2.85; 95% CI 0.17-48.86), low birth weight (OR=4.99; 95% CI 0.14-176.31), involvement of temporal lesion (OR=4.63; 95% CI 0.72-29.86) were no longer significant (Table-III).

**Table-II:** Factors for preoperative seizure due to CMs in central nervous system (52 cases).

*Features*	*E-Group(n=31)*	*NE-Group(n=21)*	*p value*
Mean age at onset	31.3	34.0	0.473
Family history of epilepsy	1	0	0.406
Pre-, peri- and post-natal factors	14	3	0.020
Complication of pregnancy	1	0	0.406
Low gestational age	3	0	0.142
Low birth weight	10	1	0.017
Complication of delivery	1	1	0.798
Febrile seizure	3	1	0.514
Psychomotor retardation	0	0	-
History of encephalitis	1	0	0.406
History of head trauma	6	1	0.130
History of operation	3	4	0.331
History of tobacoo addicion	11	7	0.873
History of alcohol addiction	10	9	0.436
Multiplevs. single lesion	2	0	0.235
Supratentorialvs. infratentorial	31	12	-
Cortical vs. subcortical	24	5	0.025
Frontal lobe	7	5	0.918
Parietal lobe	7	2	0.222
Temporal lobe	17	3	0.003
deep brain area	0	3	0.030
Side (right vs. left)	14/17	6/15	0.599
Size (diameter**≥**2.5cm)	12	10	0.084

**Table-III:** Logistic regression analysis of risk factors for seizure due to CMs

*Risk factors*	*Odds ratio*	*95% CI*	*p value*
Pre-, peri- and post-natal factors	2.85	0.17-48.86	0.470
Low birth weight	4.99	0.14-176.31	0.377
Temporal lobe	4.63	0.72-29.86	0.107
Cortical involvement	10.48	1.61-68.23	0.014

***Factors associated with postoperative seizures: ***There were 21 cases had clearly clarified the data of size of CMs during surgical process. Intraoperative electrocorticography (ECoG) was performed in 19 cases and additional resection was performed as indicated by the ECoG findings. Follow-up information of 46 (88.5%, 46/52) patients was collected while the other 6 patients were lost to follow-up (4 of E-Group and 2 of NE-Group) (Table-IV). "The mean follow-up time was 24.8 months (range 14-41 months) in E-Group and 26.6 months (range 14-43 months) in NE-Group. In E-Group, each received regular medication after surgery following doctor’s advice. There were 20 (74.1%, 20/27) received monotherapy while 7 (25.9%, 7/27) duotherapy after surgery. Continued AEDs therapy lasted for less than 3 months in 7 out of 20 patients on monotherapy while the other 20 patients went on medication for a longer period of time. At our final follow-up, 19 (70.4%, 19/27) patients had withdrawal medication. Most (77.8%, 21/27) patients were seizure free and classified as Engel class I while the other 6 (22.2%, 6/27) patients still had seizure now or then. In NE-Group, 18 (94.1%, 18/19) patients in total got symptom-free. In our study, no mortality occurred.

**Table-IV:** Postoperative follow-up outcome of 44 patients with CMs

*Group*	*No. of patients*	*Signs and symptoms, n (%)*				*Mean follow-up,* *month, (range)*
		*Engel I or free*	*Engel II or improvement*	*Engel III or no change*	*Engel IV or Worsen*	
E-Group	27	21(77.8)	5(18.5)	0	1(3.7)	24.8(14-41)
NE-Group	19	18(94.7)	0	0	1(5.3)	26.6(14-43)
Total	46	37(84.1)	5(11.4)	0	2(4.5)	25.5(14-43)

Patients with temporal lobe CMs were found more likely to be seizure free after surgery (*p*=0.016) (Table-V). The maximum diameter of CMs longer than 2.5cm and disease course longer than one year predicatedunfavorable outcome (*p*=0.012, 0.050). Other factors such as mean age at onset, mean disease course, gender, family history of epilepsy, pre, peri-, and post-natal factors were not associated with seizure outcome. Moreover, application of ECoG,surgical strategy and postoperative medication therapy did not make significant difference to seizure outcome. Of the 8 cases where no ECoG were applied, 6 (75%, 6/8) were temporal lobe involved. In the multivariate analysis, temporal lobe involvement still showed a favorable outcome (OR=0.038; 95% CI 0.002-0.833) while the size of CMs was no longer significant predictors for seizure outcome.

**Table-V:** Factors associated with postoperative seizure of CMs

*Features*	*Seizure free * *(n=21)*	*Not seizure free* *(n=6)*	*p value*
Gender ration (M:F)	3.2	1.0	0.215
Mean age at onset, y	30.7	31.0	0.976
Disease course (≥1y vs.<1y)	12/9	6/15	0.050
Family history of epilepsy	1	0	0.586
Pre-, peri- and post-natal factors	11	2	0.410
Multiple vs. single lesions	2	0	0.432
Frontal lobe	4	3	0.127
Parietal lobe	3	2	0.289
Temporal lobe	15	1	0.016
Cortical vs. subcortical	16	5	0.711
Side (right vs. left)	8	3	0.601
Size (diameter ≥2.5cm vs. not)	6/15	6/0	0.012
Seizure type(generalized vs. partial)	16/5	3/3	0.215
Total vs. subtotal resection	20/1	5/1	0.326
Perilesional hemosiderin removal	9	3	0.756
Intraoperative ECoG	14	5	0.430
Monotherapy vs. duotherapy	15/6	5/1	0.557

**Table-VI:** Logistic regression analysis of risk factors for postoperative seizure

*Risk factors*	*Odds ratio*	*95% CI*	*pvalue*
Temporal lobe	0.040	0.002-0.833	0.038
Size (diameter ≥2.5cm)	<0.001	-	0.999

## DISCUSSION

Cavernous malformations (CMs) are common vascular malformations in the central nervous system which usually consist of enlarged thin-wall blood vessels without neural tissue. The prevalence of CMs in population is around 0.4-0.8% and epileptic seizures are common clinical presentation of patients with CMs (23-50% of cases).^1^ It is reported that the risk for seizure development was about 1.51% per patient year, and 2.48% per lesion year in multiple lesion.^6^Few studies considered about the specific risk factors for developing epileptic seizure in patients with CMs especially preoperative.In this study, we discussed about the clinical characteristics and factors associated with preoperativeepileptic seizuresand surgical outcome.

In this study, 52 patients with CMs underwent surgery were included at a mean age of 36 years old. About 59.6% of patients presented preoperative epileptic seizure and the predominant seizure type was secondarily generalized seizure which was in accordance with previous studies.^7^^,^^8^Cortical involvement, temporal lobe and low birth weight were found in tight association with preoperative epilepsy in our study while cortical involvement increase this risk by 10.5 times in multivariate analysis. It was in accordance with those reported by Leone and his colleagues.^5^ However, Brunon et al found out that epileptic seizure is frequently correlated to frontal lobe which was not the situation in our study.^9^It might attribute to difference in sample size, region and race. CMs may occur as solitary (80%) or multiple lesions and the latter was often reported to increase risk for epilepsy.^9^^,^^10^ However, it could not be accurately measured because there were only 2 such cases in our study. As the limitation of sample size, low birth weight and other pre-, peri- and post-natal factors were different among E-Group and NE-Group but were not significant in multivariate analysis.

Patients with seizures caused by CMs usually achieved seizure control by antiepileptic drugs in about 60% while surgical resection seemed to be a superior method with satisfactory outcome (symptom free or Engel I) after surgical in up to 80% of all patients. However,factors associated with surgical outcome remains controversial.^11^^-^^13^ In our study, 77.8% epileptic patients achieved seizure free after surgery. Temporal lobe involvement and maximum diameter of lesion less than 2.5 cm predicted better outcome and this finding was in accordance with those reported by Baumann.^11^This might suggest that CMs located in different site may have different type of functional connectivity and involvement with surroundingbrain area to generalize epileptic discharge. However, it needs further physiological and functional investigations.

Several studies have showed patients with shorter epileptic duration and lower number of preoperative seizures were associated with better seizure control.^14^A lack of seizure control might be due to poor surgical strategy such as subtotal resection or persistence of surrounding hemosiderin deposits.^8^^,^^11^^,^^15^ However, several studies had demonstrated pure lesionectomy might eliminate epileptic seizure.^14^^,^^16^ This might attribute to the pathological differentiation characteristicof CMs which are commonly well-circumscribed lesions.It was in accordance with our findings which showed no significant difference between the different surgical strategies.It is still controversial whether the pure lesionectomy is acceptable to achieve satisfactory surgical outcome and further studies with larger sample sizeis needed.

Intraoperative ECoG is a commonly used technique in epilepsy surgery to detect epileptic discharge and help identifying the epileptogenic zone. However, due to its own limitations such as short duration, lack of ictal records and anesthesia disturbance, its application in epileptic surgery is controversial. In our study, we found no significant association between application of ECoG and postoperative seizure occurrence as reported before.^11^^.^^17^In the 8 (25.8%, 8/31) cases who had achieved favorable seizure outcome without applying ECoG, most were temporal lobe involved and this may explain the results. The ratio of ECoG usage is 74.2% (23/31) in our hospital considering epileptic patients due to CMs and it is much lower than those of hospitals in developed countries and areas partly due to financial status of patients.^17^^,^^18^

Continued AEDs treatment after surgery is still controversial. Ferroli et al found that antiepileptic drugs were not necessary in most cases and part of patients with long clinical history of seizures might need continued AEDs.^19^In our study, all patients received AEDs postoperatively and there were about 30% patients on medication at final follow up. Parts of patients with CMs might require AEDs after surgery so that it might be reasonable to prescribe medicine for patients especially with high risk for postoperative seizures.

Our study has several limitations. The primary limitation was its retrospective nature which might lead to a possible selection bias. Another limitation was limited sample size. However, as relatively low incidence of CMs with presentation of seizure, it is sometimes reasonable to explore related factors through retrospective study. The follow-up was not long. All these factors should be considered in the future studies.

## CONCLUSION

Our study suggests that the cortical involvement is the risk factors for preoperative epilepsy in patients with CMs. Surgical treatment of patient with CMs is satisfactory in most cases and temporal lobe involvement usually predict favorable postoperative seizure outcome whether under the monitoring of ECoG or not. Continued AEDs were recommended after successful surgical resection. Thus, epileptic patients with CMs should be considered for surgical treatment especially when cortical brain layer or temporal lobe was involved.
